# Epithelioid hemangioma (angiolymphoid hyperplasia with eosinophilia) of the orbit: a case report

**DOI:** 10.1186/1752-1947-1-30

**Published:** 2007-06-25

**Authors:** Bruno F Fernandes, Abdullah Al-Mujaini, Tina Petrogiannis-Haliotis, Abdullah Al-Kandari, Bryan Arthurs, Miguel N Burnier

**Affiliations:** 1 Department of Ophthalmology and Pathology. Henry C. Witelson Ocular Pathology Laboratory & Mcgill University Health Centre, Montreal, QC, Canada; 2 Department of Ophthalmology. Federal University of São Paulo, São Paulo, Brazil; 3 Department of Ophthalmology Mcgill University Health Centre. Montreal, QC, Canada; 4 Department of Pathology. Sir Mortimer B. Davis – Jewish General Hospital. Montreal, QC, Canada

## Abstract

**Background:**

Angiolymphoid hyperplasia with eosinophilia (ALHE) and Kimura's Disease (KD) share many clinical and histopathological features. Although they were once considered different stages of the same disease, they are now known to represent separate entities. Recently, ALHE is being called epithelioid hemangioma (EH), a term that better describes the possible neoplastic nature of the entity.

**Case Presentation:**

An eighteen year-old Asian female presented with a three-month history of fluctuating swelling and ptosis of the left upper eyelid. Computed tomography disclosed a distinct homogeneous lesion in the left superior orbit, molding to the globe and other orbital structures. At histopathological evaluation the lesion was composed of numerous blood vessels lined by plump endothelial cells with oval nuclei protruding into the lumen. Surrounding the vessels, there was a chronic inflammatory infiltrate with a large proportion of eosinophils. Based on clinical and histopathological findings, the diagnosis of EH was made.

**Conclusion:**

Although exams like blood count, urinalysis and whole body scans can assist in the differential diagnosis, EH can be diagnosed and differentiated from KD on histopathological grounds. The presence of vascular hyperplasia with plump endothelial cells protruding into the lumen is the most important discriminator in establishing the diagnosis of EH. Such distinction is crucial for the patient because EH is not associated with any of the systemic manifestations present in KD.

## Background

Angiolymphoid hyperplasia with eosinophilia (ALHE) and Kimura's Disease (KD) share many clinical and histopathological features. [1] Although they were once considered different stages of the same disease, they are now known to represent separate entities. [2] Recently, ALHE is being called epithelioid hemangioma (EH), a term that better describes the most distinguish feature of this entity: the abnormal proliferation of endothelial cells. [3]

EH usually presents as small, red, pruritic plaques in the subcutis or dermis of the head and neck region. Orbital involvement in EH is a relatively rare manifestation of the disease with only scattered case reports published in literature. [4]

## Case Presentation

**Figure 1:**
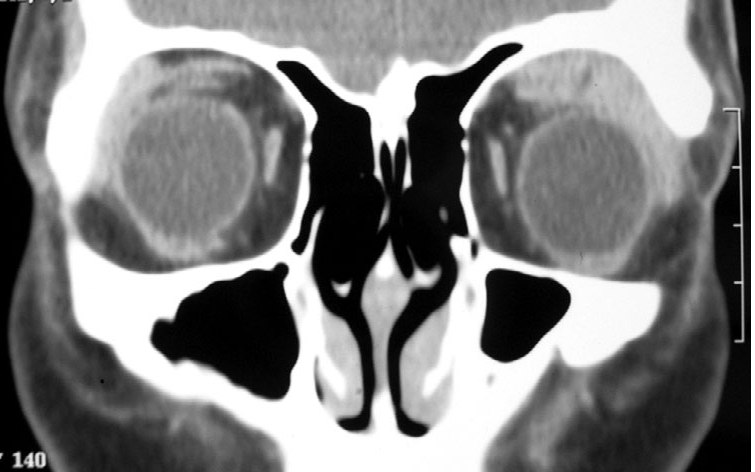
Computerized Tomography. A homogeneous lesion in the left superior orbit, molding to the globe and other orbital structures.

An eighteen year-old Asian female presented to the ophthalmology clinic of the McGill University Health Center with a three-month history of fluctuating swelling and ptosis of the left upper eyelid. Mild discomfort was felt whenever the swelling was more intense. A well-defined, soft lesion in the left upper eyelid could be palpated, just below the superior orbital rim, without associated inflammatory signs. No decrease in visual acuity or alterations of extraocular movements was found. Intraocular pressure was 17 mmHg OD and 20 mmHg OS. Computed tomography disclosed a distinct homogeneous lesion in the left superior orbit, molding to the globe and other orbital structures (Fig. 1). There was no bone erosion. The findings favored the diagnosis of a lymphoid lesion and a transpalpebral biopsy was indicated and performed.

**Figure 2:**
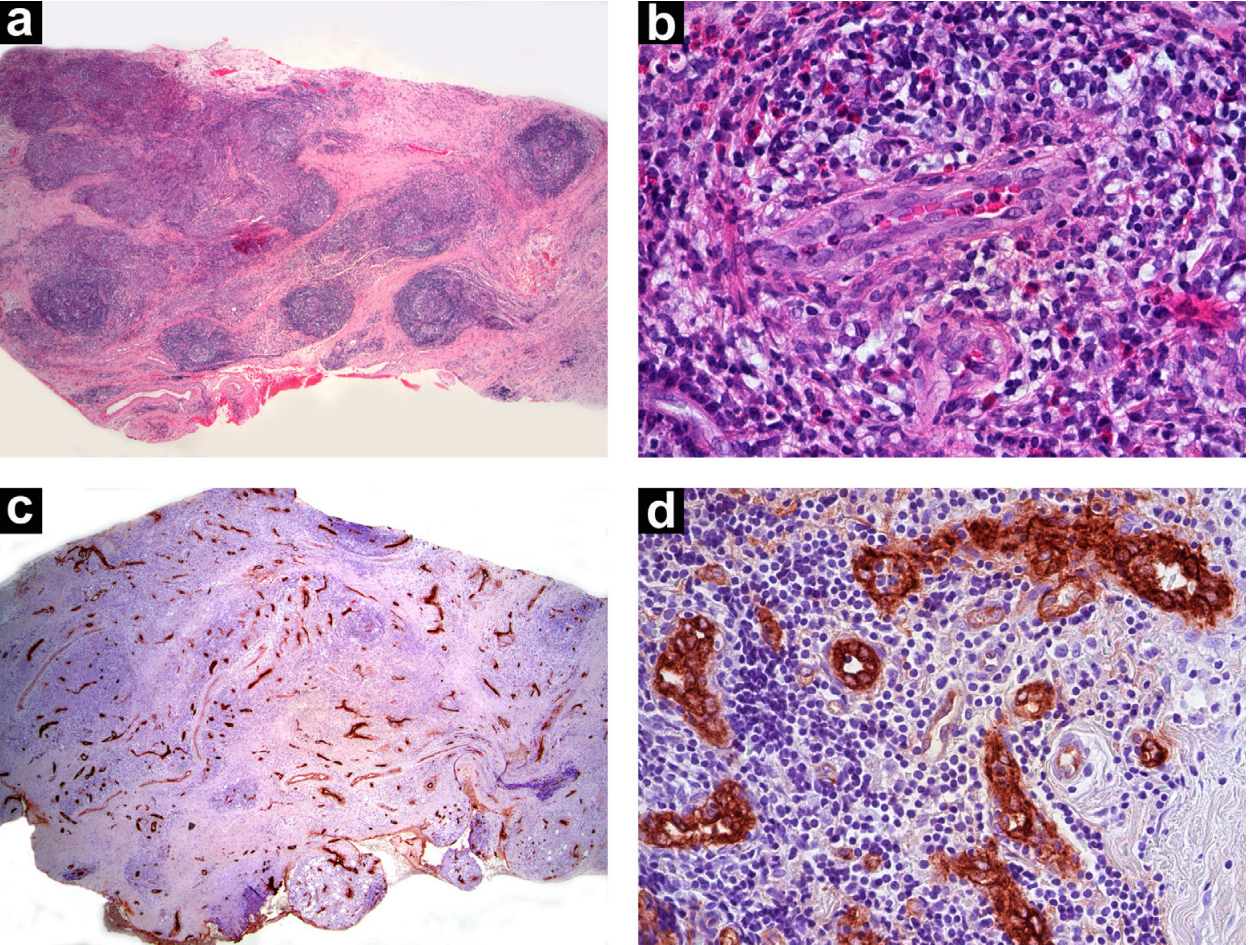
A) Low-power photomicrography showing structures resembling lymphoid follicles surrounded by loose connective tissue (H&E; original magnification × 25). B) Plump endothelial cells, surrounded by an inflammatory infiltrate containing a large number of eosinophils (H&E; original magnification × 400). C) Factor VIII immunostaining, highlighting the florid vascular proliferation (Original magnification × 25). D) The atypical endothelial cells all stained positive (Factor VIII, original magnification × 400).

Histopathological evaluation revealed the presence of structures resembling lymphoid follicles surrounded by loose connective tissue (Fig. 2A). At higher magnification, those structures were composed of numerous blood vessels lined by plump endothelial cells with oval nuclei protruding into the lumen (Fig. 2B). Surrounding the vessels, there was a chronic inflammatory infiltrate composed of lymphocytes, plasma cells and a large proportion of eosinophils. Immunohistochemical studies, performed on paraffin-embedded tissue, showed the following: Factor VIII underscored the marked vascularity of the lesion (Fig. 2C), highlighting atypical vascular lining with "epithelioid" or "histiocytoid" cells (Fig. 2D). Whole body gallium scan failed to reveal lymph node involvement elsewhere. Blood counts and urinalysis were normal. Based on clinical and histopathological findings, the diagnosis of EH was made.

## Conclusion

Epithelioid hemangioma (EH) and Kimura's Disease (KD) share many clinical and histopathological features [1] Although they were once considered different stages of the same disease, they are now known to represent separate entities [2] The former is a localized hyperplasia of atypical endothelial cells with no systemic involvement. On the other hand, the latter can course with lymphadenopathy, blood eosinophilia, and nephrotic syndrome due to IgE deposition in the renal glomeruli [1]

EH was first described in 1969 [5] It presents as nodules or erythematous subcutaneous papules, usually in the head and neck region of young women [6] It can occur in all races. Whenever the orbit is involved, common symptoms are proptosis, tearing, pruritus around the eye, and blurred peripheral vision. [4] The case presented herein had no associated symptoms besides the swelling of the eyelid, which makes the presentation even more atypical. Histologically, most lesions are well-circumscribed and composed of vessels lined by plump endothelial cells that protrude into the lumen in a "tombstone fashion" [7] Surrounding the vessels, there is usually a prominent inflammatory infiltrate. A large proportion of eosinophils can often be seen.

KD probably represents an allergic or autoimmune response that typically presents as subcutaneous nodules in the head and neck region of young Asian males [6] Systemic associations include blood eosinophilia, nephrotic syndrome due to IgE depostion in the renal glomeruli, lymphadenopathy and, less common, asthma, tuberculosis and Loffler syndrome.[1]

Although exams like blood count, urinalysis and whole body scans can assist in the differential diagnosis, EH can be diagnosed and differentiated from KD on histopathological grounds. The presence of vascular hyperplasia with plump endothelial cells protruding into the lumen is the most important discriminator in establishing the diagnosis of EH. Such distinction is crucial for the patient because EH is not associated with any of the systemic manifestations present in KD.

## Competing interests

The author(s) declare that they have no competing interests.

## Authors' contributions

BF, TH, AAlk and MNBJr were the pathologists that performed the histopathological evaluation. AAlm and BA are ophthalmogists from the Oculoplastics departments and were the attending physician responsible of providing all the clinical information All authors participated in the design of the manuscript. BF, AAlk and AAlm helped to draft the manuscript while TH, BA and MNBJr done the final revisions of the paper. All authors read and approved the final manuscript.
